# Physical Function, Muscle Strength, and Fatigue in Patients with Multiple Sclerosis: An Exploratory Cross-Sectional Study

**DOI:** 10.3390/jfmk10040477

**Published:** 2025-12-10

**Authors:** Olimar Leite de Assis Cunha, Luciane Coral Siciliani, Marcelo Barbosa Anzanel, Whesley Tanor Silva, Tatiana Rehder Gonçalves, Mauro Felippe Felix Mediano, Marina Papais Alvarenga, Regina Maria Papais Alvarenga, Hélcio Alvarenga Filho

**Affiliations:** 1 SARAH International Center for Neurorehabilitation and Neurosciences, Rio de Janeiro 22775-040, RJ, Brazil; olimar.cunha@gmail.com (O.L.d.A.C.); lucianecoral@hotmail.com (L.C.S.); marceloanzanello@gmail.com (M.B.A.); 2 Evandro Chagas National Institute of Infectious Diseases, Oswaldo Cruz Foundation, Rio de Janeiro 21040-360, RJ, Brazil; whesleytanor@gmail.com; 3 Department of Epidemiology, Institute of Social Medicine, State University of Rio de Janeiro, Rio de Janeiro 20550-013, RJ, Brazil; tatianarehder@gmail.com; 4 Department of Research and Education, National Institute of Cardiology, Rio de Janeiro 22240-006, RJ, Brazil; 5 Department of Neurology, Federal University of the State of Rio de Janeiro, Rio de Janeiro 21941-901, RJ, Brazil; alvarenga_marina@hotmail.com (M.P.A.); regina_alvarenga@hotmail.com (R.M.P.A.); helcio_alvarenga@hotmail.com (H.A.F.)

**Keywords:** multiple sclerosis, muscle strength, physical fitness, muscle fatigue, cross-sectional study

## Abstract

**Background**: Physical function, muscle strength, and fatigue are often impaired in patients with multiple sclerosis (MS). This study aimed to assess these parameters and their associations. **Methods**: This cross-sectional study included patients with relapsing-remitting MS. Physical function was assessed using the dynamic gait index (DGI), two-minute walk test (2MWT), and Expanded Disability Status Scale (EDSS). Muscle strength and fatigue were assessed using a load cell (measured in kgf). Generalized linear models (GLMs) with log link and gamma distribution examined the associations between MS and physical function, muscle strength, and fatigue. In the MS group, GLMs explored links between fatigue, muscle strength, and physical function. **Results**: Forty-seven individuals participated (18 MS; 27 controls). Patients with MS showed reduced physical function and muscle strength, and higher fatigue. Knee extension fatigue was associated with DGI (Exp β = 0.23; *p* = 0.03), 2MWT (Exp β = 0.11; *p* = 0.02), and EDSS (Exp β = 17.17; *p* < 0.0001); knee flexion fatigue was associated with EDSS (Exp β = 2.45; *p* = 0.006). Knee flexion and extension strength were also associated with EDSS. **Conclusions**: Patients with MS show reduced physical function and strength, increased fatigue, and knee muscle performance. The associations between strength, fatigue, and functional outcomes varied in magnitude, with knee-related measures, especially knee extension fatigue, showing the most consistent relationships.

## 1. Introduction

Multiple sclerosis (MS) is a neurodegenerative, chronic and progressive autoimmune inflammatory disease of the central nervous system that causes the destruction of myelin sheath, oligodendrocytes and axons [1]. The prevalence of MS has been increasing worldwide over the last decades, with estimates in Brazil from 14.5 cases per 100,000 inhabitants [2,3]. The main neurological consequence of MS is motor dysfunction that can affect one or more limbs, leading to disability [4]. Muscle weakness and fatigue are frequently reported in MS and potentially associated with decreased physical function [5]. Fatigue in MS is frequently investigated because it extends beyond neuromuscular impairments, presenting as a complex, multifactorial symptom encompassing both neuromuscular and perceptual components. It manifests as an overwhelming sense of tiredness, lack of energy, or exhaustion, often disproportionate to exerted effort. Additionally, fatigue exhibits significant associations with psychosocial and behavioral factors, including depression and sleep disorders [6,7]. These psychosocial factors may contribute to the substantial discrepancies often observed between fatigue measured through objective and subjective methods in MS. Importantly, subjective and objective fatigue often show limited correlation, which reinforces the need for comprehensive assessments that include both types of measures [8]. Conversely, physical function consists of the ability to perform basic and instrumental activities of daily living. This ability is often assessed by tools that reflect the demands of daily living, such as aerobic capacity while walking, balance, the ability to avoid obstacles while walking, and climbing stairs, among others [9,10].

The literature has reported a high burden of muscle weakness, fatigue and physical deconditioning among patients with MS [11], supported by a substantial body of preclinical evidence establishing mechanisms underlying their relationship [12]. However, despite the existing mechanistic theoretical framework, evidence regarding the association between physical function with muscle strength and fatigue remains inconclusive, with some studies indicating a lack of association between these variables due to factors such as high heterogeneity in methods (methodological heterogeneity) and in results (statistical heterogeneity) [6,13]. For instance, Rooney et al. (2019) [13] found a small effect size and high heterogeneity in their meta-analysis, with five out of seven studies showing no significant association between muscle strength and aerobic capacity. Similarly, when evaluating the association between fatigue and aerobic capacity, there was a moderate effect size (r = −0.471; 95% CI −0.644 to −0.251; *p* < 0.001), but with high heterogeneity across studies (I^2^ = 70.18%) [12]. In another review, Ramari et al. (2020) [14] found that muscle strength explained a significant portion (20–30%) of the variance associated with functional testing. Despite these findings, the strength and consistency of the relationships among subjective fatigue measures, muscle strength assessments, and physical function outcomes remain unclear and warrant further investigation [15].

While some evidence supports an association between objective aspects and physical function, the relationship that considers both objective and subjective aspects in relation to physical function is still inconclusive [15]. The limited knowledge about the factors associated with physical function can make difficult the development of effective interventions, missed opportunities for early prevention, and consequently inadequate clinical decision-making [16]. Recent studies have further highlighted the uncertainty and complexity of the relationship between fatigue and function/disability in MS. For example, Ezzeldin et al. (2023) [17] identified the Expanded Disability Status Scale (EDSS) and infratentorial white matter lesion volume as significant predictors of fatigue severity in relapsing-remitting MS. Conversely, Luostarinen et al. (2023) [18] found that, despite a moderately strong correlation between fatigue and EDSS, there was no association between EDSS and accelerometer-measured physical activity or MS functional scores. These findings reinforce that the relationship between objective and subjective fatigue and physical function is not fully understood and requires further investigation.

The controversy across studies in literature may be attributed to the reliance on subjective assessment methods in most studies, which can introduce measurement error. Therefore, studies utilizing objective measurements are needed to clarify the relationship between these variables [19]. The hypothesis of this study is that both muscle strength and fatigue are associated with physical function in patients with MS, given the demand for muscular strength and resistance to fatigue during the execution of tasks that determine physical functioning. In this context, the present study aims to assess objective and subjective physical function, muscle strength, and fatigue in individuals with MS, and to determine how these measures differ from those in individuals without MS. Additionally, within the MS group, we examined the associations of muscle strength and fatigue with physical function.

## 2. Materials and Methods

### 2.1. Study Design and Participants

This is a cross-sectional study including clinically stable patients diagnosed with relapsing-remitting MS of both sexes, aging > 18 years, and receiving standard clinical therapy at SARAH Network of Rehabilitation Hospitals, Brazil. Based on a review of medical records, patients with clinical complications or contraindications to physical exercise, those with unstable neurological condition over the last six months (defined as relapses or clinical evidence of neurological changes), those with musculoskeletal injuries that preclude the performance of physical tests, those with psychiatric disorders, active rheumatic diseases, acute infections, or other clinical causes of fatigue were excluded from the study. A control group comprising healthy participants matched (hospital workers) by age and sex was also included to compare measurements of physical function, muscle strength, and fatigue.

### 2.2. Measurements

Clinical and laboratory data were collected from medical records of patients with MS. In addition, patients with MS and controls performed tests of physical function, muscle strength, and fatigue under the supervision of previously trained staff. All tests were conducted on the same day in the afternoon, with at least two hours between assessments.

#### Physical Function (PF)

PF was evaluated by the two-minute walk test (2MWT), dynamic gait index (DGI), and Expanded Disability Status Scale (EDSS).

### 2.3. Two Minute Walk Test (2MWT)

The 2MWT evaluates the maximum distance traveled during two minutes in a course of 30 m, on a smooth and flat floor. At the start signal, participants were encouraged to walk as fast as possible, without running, in the demarcated course area, during 2 min. Participants could pause for rest on the course, if necessary. Standardized encouragement was provided by the evaluator every 30 s. The test would be discontinued if participants exhibited signs of dizziness, pain, nausea, and excessive fatigue [20]. The test is widely used and validated for evaluating people with MS [21].

### 2.4. Dynamic Gait Index (DGI)

The DGI assesses balance changes through a series of eight functional tasks, including variations in gait speed, head movements, pivot turns, obstacle navigation, and stair climbing. For the application of the test, two plastic cones of 50 cm high and a step bank with 40 cm long, 20 cm wide, and 15 cm high were used. The eight tasks to be performed were: (1) walking on a level surface, (2) changing gait speed, (3) walking with horizontal head turns, (4) walking with vertical head turns, (5) pivot turn, (6) stepping over an obstacle, (7) stepping around obstacles, and (8) stair climbing. Each task was scored from 0 to 3 (3 = Normal gait, 2 = mild impairment, 1 = moderate impairment and 0 = severe impairment), with a maximum total score of 24 points; lower scores indicate worse performance [22,23]. The test was administered by a certified physiotherapist experienced in evaluating patients with MS.

### 2.5. Expanded Disability Status Scale (EDSS)

The EDSS, a widely used tool for quantifying disability in people with MS, was applied only to participants with MS. The scale evaluates dysfunction across various neurological functional systems including pyramidal, cerebellar, sensory, brainstem, sphincter, visual, and mental domains. Each functional system is scored individually on a scale from 0 (no impairment) to 6 (severe impairment). To determine the overall EDSS score, an algorithm integrates the individual functional system scores along with the patient’s mobility status and daily activity limitations. The final disability score ranges from 0.0 (normal neurological function) to 10.0 (death due to MS-related complications) [24].

### 2.6. Muscle Strength Assessment

Muscle strength was measured using a load cell developed and manufactured by the SARAH Network of Rehabilitation Hospitals (Model Alfa Instruments, São Paulo—Brazil). The procedures, including protocols, execution methods, and muscle groups tested, were standardized [25,26], and strength measurements were presented in kgf. After a familiarization protocol, consisting of a 5 s submaximal isometric contraction for each muscle group, participants performed tests lasting 5 and 30 s to assess the maximum voluntary isometric strength. First, the test was performed with a 5 s contraction, followed by a 5 min rest to avoid any interference due to previous fatigue, after which the 30 s contraction test was initiated [27]. Given that the study was conducted in a clinical setting with a continuous flow of patients, and in accordance with the unit’s clinical protocol, only the right limb of each participant was evaluated to ensure consistency, reduce testing time, and prevent excessive fatigue. Standardized verbal encouragement was provided throughout the assessments. All tests were performed by the same evaluator, and muscle groups (knee and elbow flexors and extensors) were tested in a defined order, alternating between lower and upper limbs to allow for recovery intervals. A detailed description of the instrument and assessment is available in the Supplementary Materials.

### 2.7. Fatigue Assessment

Fatigue was assessed through both direct measurements and specific scales.

### 2.8. Motor Fatigue Index (MFI)

The MFI assesses fatigue by measuring muscle contraction sustained for 30 s [23]. MFI calculations were performed for the elbow and knee flexors and extensors using a load cell. This index was determined based on the area under curve (AUC) of force vs. time, as previously described [25,26]. Further details of the calculation are provided in the Supplementary Materials. The value used for the muscle strength assessment was the maximum force (F_max0–5_) reached during the 5 s contraction. This maximum value was used both as the measure of maximal voluntary contraction (MVC) and as a reference point for calculating the motor fatigue index (MFI), according to the formula MFI = 100% × [1 − (AUC_5–30_/(F_max0–5_ × 25))].

### 2.9. Fatigue Severity Scale (FSS)

The FSS consists of nine items that evaluate the severity of fatigue symptoms and their impact on daily activities over the past two weeks. Each item is rated on a 7-point scale (1 to 7). The total score is calculated by summing the individual item scores, ranging from 9 to 63, with higher scores indicating greater fatigue [28].

### 2.10. Modified Fatigue Impact Scale (MFIS)

The MFIS is a 21-item questionnaire designed to assess the impact of fatigue across three domains: physical, cognitive and social. Each item is rated on a scale from 0 to 4. The total score is calculated by summing the points from all three domains, ranging from 0 to 84, with higher scores indicating greater fatigue [28].

### 2.11. Covariates

Covariates included age, sex, and depressive symptoms. Depressive symptoms were assessed using the Beck Depression Inventory (BDI) [29], a 21-item self-report questionnaire. Each item consists of four response options scored from 0 to 3, with the total score calculated as the sum of all item scores, ranging from 0 to 63. Higher scores indicate greater levels of depression [29].

### 2.12. Data Analysis

Data normality was assessed using the Skewness and Kurtosis test, an implementation of the D’Agostino–Pearson omnibus test. Due to the asymmetric distribution, continuous variables were expressed as median and 25–75% interquartile range. Categorical variables were presented as absolute numbers and percentages. Comparison of characteristics between MS participants and the control group were performed using the Mann–Whitney test for continuous and chi-squared test for categorical variables. Effect sizes were calculated using Cohen’s r. We used a Spearman’s Rho correlation analysis to explore the relationships between the different fatigue measures, presenting the results in a correlation matrix. Generalized linear models (GLMs) with log link and gamma distribution, which are appropriate for modeling skewed, non-normally distributed continuous outcomes, were fitted to examine the association between the MS diagnosis (exposure) and physical function, muscle strength, and fatigue (outcomes). Models were adjusted for potential confounders (age, sex, and depressive symptoms). In addition, considering only MS participants, we explored the association between fatigue and muscle strength (exposures) with physical function (outcome) also using GLMs with log link and gamma distribution, adjusted for the potential confounders age, sex, and depressive symptoms. The beta coefficients of GLMs were exponentiated (Exp β) to facilitate the interpretation of the results. The exponentiated beta coefficients (Exp β) from GLMs represent the proportional change in the outcome for each one-unit increase in a continuous exposure or change in category of a categorical variable: values equal to 1 indicate no change; values less than 1 indicate a percentage decrease; and values greater than 1 indicate a percentage increase in the outcome. Corrected *p*-values were included in the manuscript using the Bonferroni method (i.e., multiplying the uncorrected *p*-value by the number of comparisons) to enhance transparency and allow readers to assess the robustness of the findings, particularly in borderline cases. This method is intentionally conservative to control type I error, although it may increase the risk of Type 2 errors, especially when multiple comparisons are involved. The analysis of Variance Inflation Factors (VIF) indicated no evidence of multicollinearity (VIF = 1.07). No evidence of overdispersion was observed, as the ratio of residual deviance to residual degrees of freedom was close to 1. Primary analyses, including descriptive statistics, *t*-tests, and GLMs, were performed using Stata 17.0. Correlation analyses between fatigue measures, as well as the creation of graphical representations, were conducted using R, version 4.4.3.

## 3. Results

In total, 47 individuals participated in the study (18 with MS and 27 controls without the disease). Participants’ characteristics are presented in Table 1. Overall, individuals with MS demonstrated higher fatigue levels and reduced muscle strength and physical function compared to those without the disease. The EDSS scores in the MS group ranged from 2.0 to 6.5, with a median score of 4.0. Most participants (≈60%) with MS had EDSS scores between 2 and 4, indicating preserved mobility with some functional limitations, as shown in Figure S1 of the Supplementary Materials.

**Figure 1:**
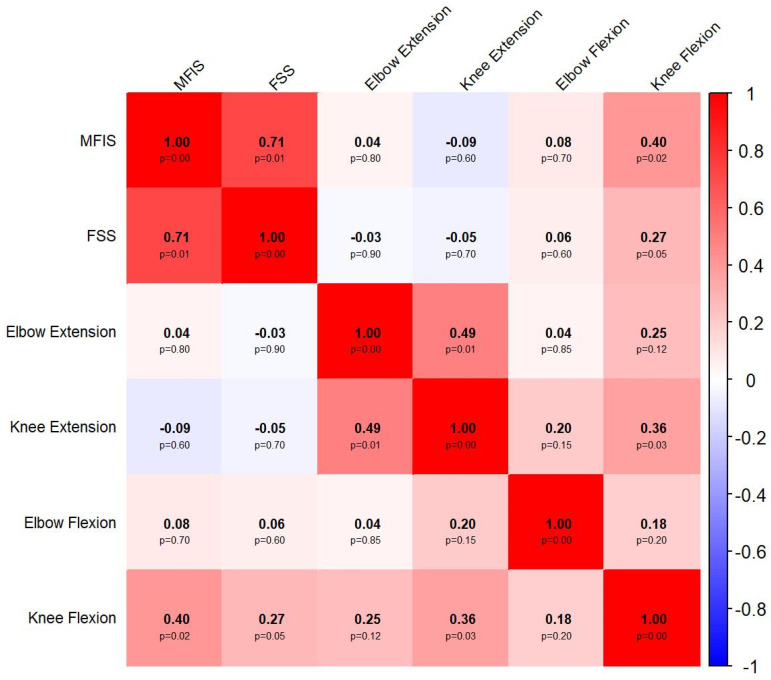
Correlations between the different measures of fatigue. Abbreviations: FSS: Fatigue Severity Scale; MFIS: Modified Fatigue Impact Scale; *p*: statistical significance value.

The correlations between the different measures of fatigue are presented in Figure 1. Overall, there were strong correlations between the subjective measures [MFIS and FSS (r = 0.73, *p* = 0.01)], and moderate correlations among the objective measures [elbow extension and knee extension (r = 0.42, *p* = 0.01), as well as knee extension and flexion (r = 0.34, *p* = 0.03)]. There were no statistically significant correlations between the objective and subjective measures.

Table 2 presents the association between physical function, muscle strength, and fatigue with the presence of MS. Overall, MS was associated with reduced physical function, increased fatigue (except for elbow extension/flexion and knee extension), and decreased muscle strength.

Table 3 presented the associations of muscle strength and fatigue with physical function in patients with MS. DGI and distance walked in the 2MWT were associated with knee extension fatigue index. Conversely, the EDSS was associated with knee extension fatigue and knee flexion fatigue (fatigue assessment variables) as well as knee flexion strength and knee extension strength (strength assessment variables).

## 4. Discussion

The main findings of the present study are as follows: (1) Subjective fatigue scales (MFIS and FSS) showed a strong correlation with each other, while objective strength measures demonstrated moderate correlations among themselves, particularly between knee and elbow extension; (2) MS was significantly associated with reduced physical function and muscle strength, as well as increased in fatigue indices; (3) Among the objective fatigue measures, the knee extension fatigue index was consistently associated with all physical function measures (DGI, 2MWT, and EDSS), while the knee flexion fatigue index was specifically associated with EDSS scores; (4) only knee flexion strength (both 5 and 30 s measure) showed an inverse association with EDSS scores, with no significant association observed with the DGI and the 2MWT.

### 4.1. Associations Between Objective and Subjective Measures of Fatigue

The MFIS was moderately correlated with the FSS, and only MFIS showed a significant association with one of the objective fatigue measures (knee flexion). In parallel, objective fatigue indices also showed moderate correlations among themselves, such as between knee extension and elbow extension, and between knee extension and knee flexion. These findings support the conceptual distinction between objective and subjective fatigue [15].

### 4.2. Association of MS with Physical Function, Muscle Strength, and Fatigue

The association between MS and impaired physical function, muscle strength, and fatigue was expected, as inflammation, demyelination, axonal damage, and axonal loss may contribute to motor neuron insufficiency or inefficiency [30]. These factors reduce the capacity for muscle contraction and, consequently, the ability to generate torque (force). Additionally, individuals with MS often exhibit muscle and neuromuscular junction alterations, such as decreased oxidative capacity of muscle fibers and metabolite accumulation, contributing to fatigue [19]. Similar findings have been reported in previous studies such as Valet et al. (2017) [11], who demonstrated that fatigue is associated with walking ability in the 2MWT. Their study also identified a link between fatigue and overall mobility, emphasizing the importance of assessing these variables and their impact on daily living [11].

### 4.3. Association Between Fatigue and Strength with Physical Function in MS

Our study found that subjective fatigue assessment measurements correlate with each other, as do objective measures. This finding corroborates a previous meta-analysis [13], which reported consistent associations for subjective measures (specifically the FSS and MFIS) and for objective measures (lower limbs) with physical function when analyzed separately. In other words, there was consistency in the results when only subjective or objective measures were used, indicating that this distinction can be a major source of heterogeneity.

In patients with MS, knee extension fatigue was associated with all components of physical function assessment, while knee flexion fatigue was associated with the EDSS score. These findings can be attributed to the involvement of these large muscle groups in activities essential for physical function tests, such as walking distance and dynamic gait. Beyond fatigue, knee muscle strength, particularly knee flexion strength, has been recognized as a key marker of decline and disease progression in other conditions [31,32,33]. Our study highlights the cross-sectional relationship between the functioning of these muscles and crucial functional outcomes, corroborating previous investigations [34]. However, further studies are needed to assess the prognostic value of these measures [35].

This study provides important insights into the relationship between physical function and easily assessable, low-cost variables, contributing to a better understanding of patients’ clinical conditions. In addition, this investigation may provide a theoretical framework to support research on the effectiveness of interventions, such as physical training, and prognostic studies. However, these findings should be interpreted with caution due to certain limitations, including the small sample size and cross-sectional design, which may restrict the strength of the evidence. Sample size calculation was not performed prior to the study, and participants were sequentially recruited by convenience, which resulted in an unintentional imbalance in sample size between the healthy control and MS groups. Although this limitation may have influenced the precision of some estimates, the number of statistically significant associations observed suggests that the study likely had adequate power to detect the main relationships under investigation. Moreover, some estimates showed wide confidence intervals, which may inflate the magnitude of the effect and should therefore be interpreted with caution. The sample consisted of patients followed at a rehabilitation center, which may not be representative of the overall population with MS. The unilateral assessment of muscle strength and fatigue may introduce bias, considering the potential for asymmetries in patients with MS; therefore, this information should be considered when interpreting the results. However, because this is a study conducted in a clinical/hospital environment with a continuous flow of patients, it was decided to standardize the assessment on the dominant limb for practicality.

## 5. Conclusions

We concluded that individuals with MS exhibit lower physical function and muscle strength, along with increased fatigue, compared to those without MS. In patients with MS, knee flexor muscle strength and fatigue index, as well as knee extensor strength, were associated with physical function. Considering the exploratory nature of the present study, future research investigating longitudinal trajectories of these variables would provide a better understanding about their role in functional decline, disease progression, and facilitate the development of rehabilitation strategies. Establishing the prognostic value of these assessments through longitudinal studies will help guide evidence-based clinical practice and inform public health initiatives focused on maintaining mobility and independence for individuals with MS.

## Figures and Tables

**Table 1:** Characteristics of study participants (*n* = 47).

	Control (*n* = 29)	Patients with MS (*n* = 18)	*p*-Value	Cohen’s r
**Clinical characteristics**				
Age (years)	38 (35–41)	44.5 (37–49)	0.03	−0.32
Female (*n*, %)	14 (48.3%)	10 (55.6%)	0.63	NA
BDI	12 (3–22)	10.5 (8–16)	0.66	+0.06
**Physical function**				
Dynamic Gait Index	24 (24–24)	20 (15–23)	<0.001	+0.84
2MWT (meters)	213.5 (200.5–234)	113 (66–134.5)	<0.001	+0.82
EDSS	NA	4 (2.5–6)	NA	NA
**Fatigue**				
Modified Fatigue Impact Scale	12 (3–22)	49 (30–57)	<0.001	−0.68
Fatigue Severity Scale	27 (18–32)	46.5 (32–56)	<0.001	−0.64
Elbow Extension Fatigue Index (30 s)	0.27 (0.19–0.31)	0.27 (0.20–0.37)	0.45	−0.11
Knee Extension Fatigue Index (30 s)	0.26 (0.22–0.29)	0.24 (0.16–0.37)	0.77	−0.05
Elbow Flexion Fatigue Index (30 s)	0.24 (0.16–0.30)	0.26 (0.14–0.32)	0.82	−0.04
Knee Flexion Fatigue Index (30 s)	0.33 (0.26–0.43)	0.68 (0.38–0.86)	0.001	−0.47
**Muscle strength**				
Elbow Flexion Strength (5 s)	14.64 (10.10–19.92)	8.78 (5.44–13.91)	0.002	+0.45
Elbow Flexion Strength (30 s)	13.05 (8.99–16.87)	8.64 (6.11–11.23)	0.004	+0.41
Elbow Extension Strength (5 s)	12.48 (9.64–20.09)	8.28 (6.48–13.45)	0.007	+0.39
Elbow Extension Strength (30 s)	12.66 (8.20–17.14)	8.20 (6.53–11.22)	0.01	+0.37
Knee Flexion Strength (5 s)	12.40 (7.42–13.93)	3.27 (1.00–7.45)	<0.001	+0.66
Knee Flexion Strength (30 s)	9.58 (7.88–12.84)	2.86 (0.07–4.23)	<0.001	+0.70
Knee Extension Strength (5 s)	32.31 (27.93–51.76)	20.82 (15.41–28.34)	0.001	+0.54
Knee Extension Strength (30 s)	31.51 (24.60–39.54)	17.37 (13.85–22.17)	<0.001	+0.57

Abbreviations: 2MWT: Two Minute Walk Test; BDI: Beck Depression Inventory; NA: Not applicable—EDSS was applied only to participants with MS.

**Table 2:** Exponentiated Beta coefficients (95% CI) for the associations between multiple sclerosis with physical function, fatigue, and muscle strength (*n* = 47).

	Exp β (95%CI) *	*p*-Value (Uncorrected)	*p*-Value (Corrected) **
**Physical function**			
Dynamic Gait Index	0.76 (0.68–0.86)	<0.0001	<0.001
2MWT (meters)	0.48 (0.40–0.57)	<0.0001	<0.001
**Fatigue**			
Modified Fatigue Impact Scale	5.81 (4.25–7.95)	<0.0001	<0.001
Fatigue Severity Scale	1.83 (1.55–2.18)	<0.0001	<0.001
Elbow Extension Fatigue Index (30 s)	1.09 (0.84–1.41)	0.51	1.00
Knee Extension Fatigue Index (30 s)	1.07 (0.82–1.39)	0.63	1.00
Elbow Flexion Fatigue Index (30 s)	1.26 (0.93–1.71)	0.14	0.84
Knee Flexion Fatigue Index (30 s)	1.97 (1.47–2.63)	<0.0001	<0.001
**Muscle strength**			
Elbow Flexion Strength (5 s)	0.66 (0.55–0.79)	<0.0001	<0.001
Elbow Flexion Strength (30 s)	0.68 (0.58–081)	<0.0001	<0.001
Elbow Extension Strength (5 s)	0.72 (0.58–0.89)	0.002	0.01
Elbow Extension Strength (30 s)	0.73 (0.58–0.91)	0.005	0.04
Knee Flexion Strength (5 s)	0.33 (0.23–0.47)	<0.0001	<0.001
Knee Flexion Strength (30 s)	0.27 (0.17–0.42)	<0.0001	<0.001
Knee Extension Strength (5 s)	0.61 (0.48–0.76)	<0.0001	<0.001
Knee Extension Strength (30 s)	0.60 (0.46–0.79)	<0.0001	<0.001

Abbreviations: 2MWT: Two Minute Walk Test; * Model adjusted for age, sex, and depressive symptoms; ** *p*-value corrected for multiple comparisons (Bonferroni).

**Table 3:** Exponentiated Beta coefficients (95%CI) for the associations between muscle strength and fatigue with physical function in patients with MS (*n* = 18).

Variable	Dynamic Gait Index			2-Minute Walk Test			Expanded Disability Status Scale		
	Exp β (95%CI) *	*p*-Value (Uncorrected)	*p*-Value (Corrected) **	Exp β (95%CI) *	*p*-Value (Uncorrected)	*p*-Value (Corrected) **	Exp β (95%CI) *	*p*-Value (Uncorrected)	*p*-Value (Corrected) **
**Fatigue**									
Modified Fatigue Impact Scale	1.00 (0.99–1.01)	0.63	1.00	1.00 (0.99–1.02)	0.89	1.00	0.99 (0.98–1.01)	0.35	1.00
Fatigue Severity Scale	1.00 (0.99–1.02)	0.83	1.00	0.99 (0.97–1.01)	0.33	1.00	1.01 (0.99–1.03)	0.38	1.00
Elbow Extension Fatigue Index (30 s)	0.99 (0.22–4.36)	0.98	1.00	0.76 (0.09–6.32)	0.80	1.00	2.44 (0.40–14.97)	0.33	1.00
Knee Extension Fatigue Index (30 s)	0.23 (0.59–0.88)	0.03	0.18	0.11 (0.02–0.71)	0.02	0.12	17.17 (3.57–82.52)	<0.0001	<0.001
Elbow Flexion Fatigue Index (30 s)	0.32 (0.11–0.93)	0.04	0.24	0.34 (0.08–1.53)	0.16	0.96	2.01 (0.42–9.67)	0.39	1.00
Knee Flexion Fatigue Index (30 s)	0.74 (0.42–1.31)	0.31	1.00	0.68 (0.34–1.36)	0.27	1.00	2.45 (1.29–4.67)	0.006	0.04
**Muscle strength**									
Elbow Flexion Strength (5 s)	1.00 (0.95–1.05)	0.97	1.00	1.03 (0.96–1.10)	0.42	1.00	0.98 (0.92–1.04)	0.49	1.00
Elbow Flexion Strength (30 s)	1.05 (0.98–1.11)	0.16	1.00	1.08 (0.99–1.17)	0.07	0.42	0.94 (0.88–1.01)	0.08	0.64
Elbow Extension Strength (5 s)	1.04 (0.97–1.10)	0.26	1.00	1.06 (0.97–1.15)	0.19	1.00	0.96 (0.89–1.05)	0.39	1.00
Elbow Extension Strength (30 s)	1.01 (0.94–1.08)	0.72	1.00	1.03 (0.94–1.13)	0.48	1.00	0.98 (0.90–1.06)	0.63	1.00
Knee Flexion Strength (5 s)	1.03 (0.97–1.10)	0.38	1.00	1.03 (0.95–1.13)	0.44	1.00	0.89 (0.83–0.97)	0.007	0.05
Knee Flexion Strength (30 s)	1.06 (0.99–1.12)	0.09	0.72	1.06 (0.98–1.16)	0.15	1.00	0.87 (0.82–0.94)	<0.0001	<0.001
Knee Extension Strength (5 s)	1.00 (0.98–1.03)	0.70	1.00	1.01 (0.99–1.05)	0.33	1.00	0.98 (0.95–1.00)	0.05	0.48
Knee Extension Strength (30 s)	1.01 (0.99–1.03)	0.37	1.00	1.02 (0.99–1.05)	0.14	1.00	0.98 (0.96–0.99)	0.04	0.32

* Model adjusted for age, sex, and depressive symptoms; ** *p*-value corrected for multiple comparisons (Bonferroni).

## Data Availability

The data presented in this study are available on request from the corresponding author. The data are not publicly available due to ethical restrictions and the need to protect the privacy and confidentiality of the study participants, as data sharing was not included in the ethics committee’s approval.

## Supplementary Materials

The following supporting information can be downloaded at: https://www.mdpi.com/article/10.3390/jfmk10040477/s1, Detailed description of the instrument and assessment muscle strength assessment; Details of the Motor Fatigue Index calculation.

## Abbreviations

The following abbreviations are used in this manuscript:

Abbreviation	Meaning
MS	Multiple Sclerosis
DGI	Dynamic Gait Index
2MWT	Two-Minute Walk Test
EDSS	Expanded Disability Status Scale
GLM	Generalized Linear Model
kgf	Kilogram-force
CNS	Central Nervous System
RRMS	Relapsing-Remitting Multiple Sclerosis
