# Supplementary material for: Physical Function, Muscle Strength, and Fatigue in Patients with Multiple Sclerosis: An Exploratory Cross-Sectional Study
Source: J Funct Morphol Kinesiol. 2025 Dec 10;10(4):477. doi: 10.3390/jfmk10040477 (PMC12734181; doi:10.3390/jfmk10040477)
Supplement: Supplementary file 1 [file jfmk-10-00477-s001.zip › jfmk-3981688-supplementary.pdf]

Journal of
Functional Morphology and Kinesiology

Supplementary Material

1 Details of the Muscle Strength Assessment

Equipment description:

- The tests were carried out using a support base connected to a load cell (Model Alfa Instruments, São Paulo - Brazil), which is a force transducer that transforms a physical quantity (force) into an electric signal. The load cell was connected to a converter plate that analyzed the data and transformed them into a measure of force using software developed by the SARAH Network of Rehabilitation Hospitals team, which included exercise physiologists, engineers, and technicians (SURAKKA; ROMBERG; RUUTIAINEN; VIRTANEN *et al.*, 2004).

Detailed testing procedures:

- **Knee flexion (KF)**: To perform the tests, the participant was positioned in a standing stance with double support from the knees and trunk. The tested knee was flexed at 90°, and an adjustable heel/ankle strap was used to better distribute the load cell pressure. The volunteer was instructed to flex the knee with maximum force.
- **Elbow extension (EE)**: The participant was seated with the ipsilateral shoulder stabilized against the trunk and the elbow flexed at 90°, with the forearm in a pronated position. A stirrup was used for manual grip, and the load cell was attached to a fixed point. The volunteer was instructed to perform elbow extension with maximum force.
- **Knee extension (KE)**: The participant was seated with hips and knees flexed at 90°. An adjustable ankle strap was placed at the midpoint between the lateral and medial malleoli to better distribute the load cell pressure. The participant was instructed to perform knee extension with maximum force.
- **Elbow flexion (EF)**: The participant was seated with the ipsilateral shoulder stabilized against the trunk and the elbow flexed at 90°, with the forearm in a supinated position. A stirrup was used for hand grip, and the load cell was fixed to the ground. The participant was instructed to perform elbow flexion with maximum force.

Additional considerations:

- Volunteers were instructed to avoid strenuous activities before the assessments. Measurement reliability was ensured by assessing a known load (dumbbell) before the tests, and all measurements were performed in standardized positions.

Journal of
Functional Morphology and Kinesiology

MDPI

Figures:

KF (Knee flexion)

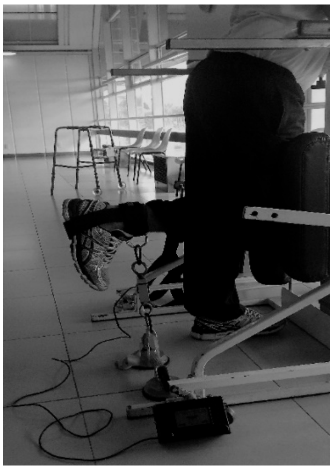

EE (Elbow extension)

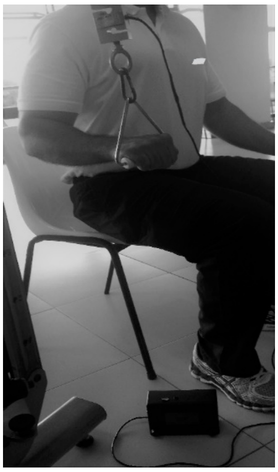

KE (Knee extension)

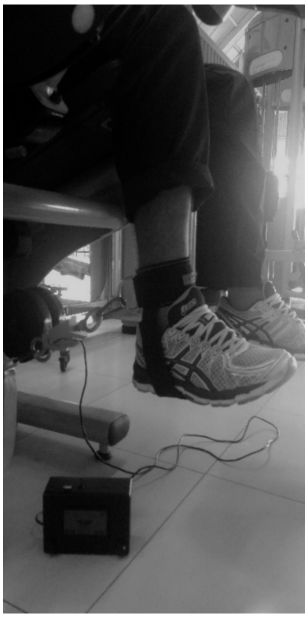

EF (Elbow flexion)

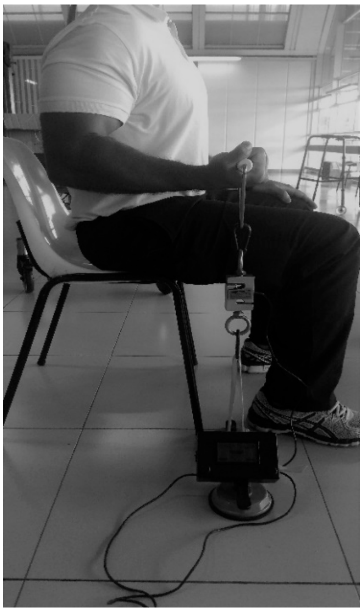

Journal of
Functional Morphology and Kinesiology

MDPI

Details of the Motor Fatigue Assessment

The motor fatigue index was calculated using the following formula: MFI = 100% × [1 – ($AUC_{5–30}$ / ($F_{max0–5}$ × 25))], where $AUC_{5–30}$ = AUC force × time for the last 25 seconds of sustained contraction, and $F_{max0–5}$ = maximum force reached during the first 5 seconds. Thus, for the evaluation, the first 5 seconds of muscle contraction (when force generation is increasing toward the peak) were excluded from the fatigue calculation. In this way, the contraction was evaluated for 30 seconds, but for the purposes of calculating fatigue, only the 25 seconds after the initial 5 seconds were considered (representing the period of peak force, not fatigue). The area under the force-time curve for the last 25 seconds of sustained contraction ($AUC_{5–30}$) was divided by a hypothetical AUC that could be produced in the absence of fatigue (i.e., if the patient sustained the same maximal force achieved during the first 5 seconds ($F_{max0–5}$) throughout the last 25 seconds). The MFI indices for KF, EE, KE, and EF were assessed.

Figure S1. Distribution of patients according to Expanded Disability Status Scale (EDSS) disease progression scores.

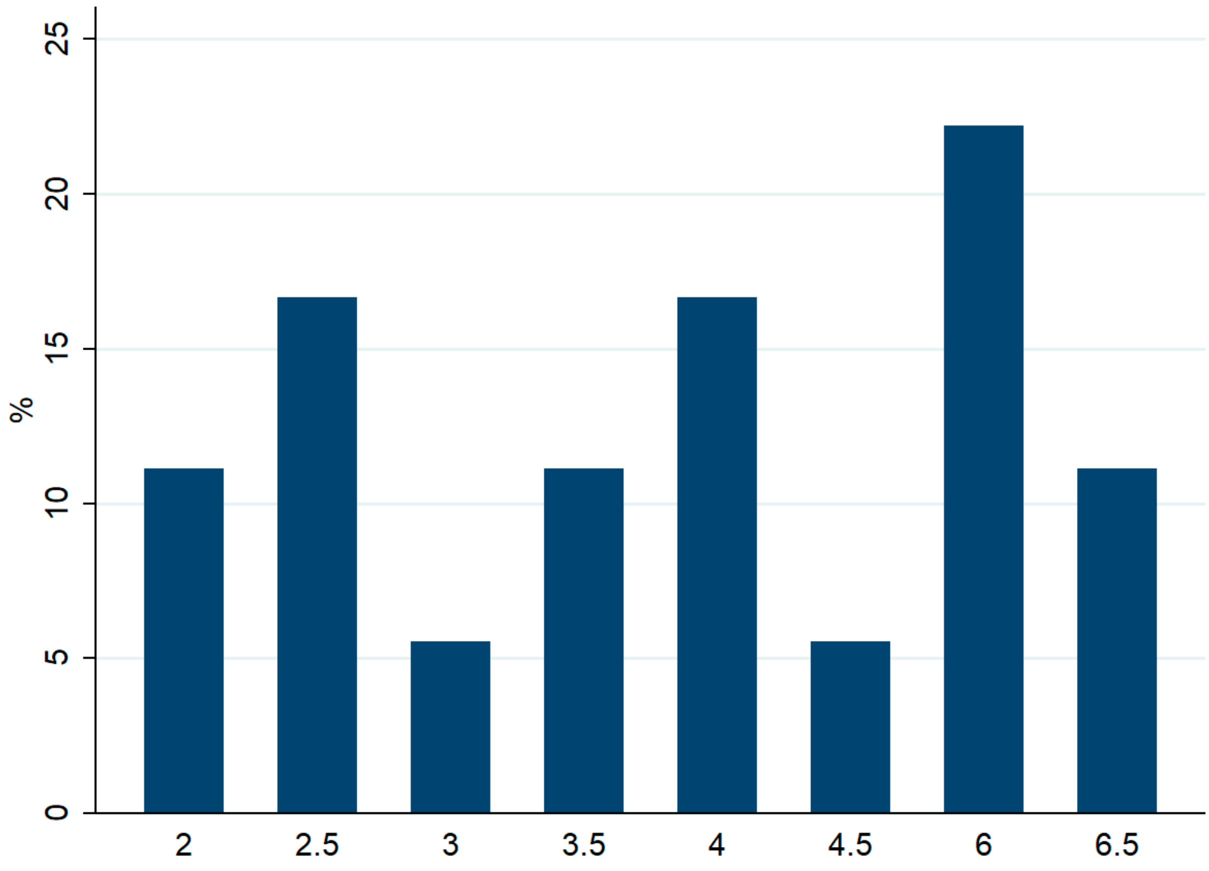

SURAKKA, J.; ROMBERG, A.; RUUTIAINEN, J.; VIRTANEN, A. *et al.* Assessment of muscle strength and motor fatigue with a knee dynamometer in subjects with multiple sclerosis: a new fatigue index. **Clinical rehabilitation**, 18, n. 6, p. 652-659, 2004.
